# Aging Compromises Terminal Differentiation Program of Cytotoxic Effector Lineage and Promotes Exhaustion in CD8 ^+^ T Cells Responding to Coronavirus Infection

**DOI:** 10.1111/acel.70109

**Published:** 2025-05-21

**Authors:** Ziang Zhu, Guohua Lou, Ying Luo, Kiddist Yihunie, Jonathan Hoar, Juan A. Daniel, Bret M. Evers, Chen Yao, Tuoqi Wu

**Affiliations:** ^1^ Department of Immunology University of Texas Southwestern Medical Center Dallas TX USA; ^2^ Immunology Ph.D. Program University of Texas Southwestern Medical Center Dallas TX USA; ^3^ Department of Immunology and Microbiology University of Colorado Anschutz Medical Campus Aurora CO USA; ^4^ Cancer Biology Ph.D. Program University of Texas Southwestern Medical Center Dallas TX USA; ^5^ Department of Internal Medicine University of Texas Southwestern Medical Center Dallas TX USA; ^6^ Department of Pathology University of Texas Southwestern Medical Center Dallas TX USA; ^7^ Kidney Cancer Program, Simmons Comprehensive Cancer Center University of Texas Southwestern Medical Center Dallas TX USA; ^8^ Harold C. Simmons Comprehensive Cancer Center, University of Texas Southwestern Medical Center Dallas TX USA; ^9^ Peter O'Donnell Jr. Brain Institute, University of Texas Southwestern Medical Center Dallas TX USA

**Keywords:** aging, CD8^+^ T cells, exhaustion, mouse hepatitis virus (MHV), terminally differentiated effector

## Abstract

T cell aging increases the risk of viral infection‐related morbidity and mortality and reduces vaccine efficacy in the elderly. A major hallmark of T cell aging is the loss of quiescence and shift toward terminal differentiation during homeostasis. However, how aging impacts the differentiation program of virus‐specific T cells during infection is unclear. Here, in a murine coronavirus (MHV) infection model with age‐associated increased mortality, we demonstrate that aging impairs, instead of promoting, the terminal differentiation program of virus‐specific CD8^+^ T cells. Upon infection, CD8^+^ and CD4^+^ T cells in old mice showed marked reduction in clonal expansion and upregulation of immune checkpoints associated with T cell exhaustion. Bulk and single‐cell transcriptomics showed that aging upregulated the T cell exhaustion transcriptional program associated with TOX in virus‐specific CD8^+^ T cells and shifted the myeloid compartment from immunostimulatory to immunosuppressive phenotype. In addition, aging downregulated the transcriptional program of terminally differentiated effector CD8^+^ T cells and diminished the CX3CR1^+^ cytotoxic effector lineage. Mechanistically, virus‐specific CD8^+^ T cells from infected aged mice displayed defects in inducing transcription factors ZEB2 and KLF2, which were required for terminal differentiation of effector CD8^+^ T cells. Together, our study shows that aging impairs terminal differentiation and promotes exhaustion of virus‐specific CD8^+^ T cells responding to coronavirus infection through dysregulating expression of lineage‐defining transcription factors.

## Graphical abstract

In a MHV infection model with age‐associated increased mortality, we demonstrate that aging impairs, instead of promoting, the terminal differentiation program of virus‐specific CD8^+^ T cells through downregulating key transcriptional regulators of terminal differentiation. In addition, aging induces the exhaustion program downstream of TOX in MHV‐specific CD8^+^ T cells.

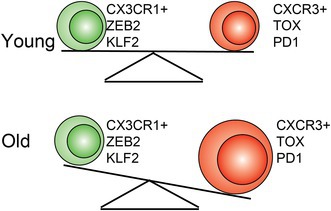

## 1 Introduction

Immunosenescence, the gradual decline of immunity with age, leads to increased infection‐related morbidity and mortality and loss of vaccine efficacy among the elderly. Most deaths associated with infections by influenza virus, SARS‐CoV, or SARS‐CoV‐2 occurred in people older than 65 (Koff and Williams 2020; Wu et al. 2021). Yet, the efficacy of most vaccines decreases significantly with age (Poland et al. 2018). Thus, understanding the mechanisms of immunosenescence in the elderly is critical for developing novel strategies to protect the aging population from infectious diseases.

T cells are essential to antiviral immunity. During most viral infections, T cells differentiate into effector T cells that express cytokines and cytotoxic proteins and mount a clonal expansion (Kaech et al. 2002). However, during chronic viral infection, antiviral T cells become dysfunctional and differentiate into exhausted T cells that upregulate immune checkpoints including PD1, progressively lose effector function, and become less proliferative (McLane et al. 2019). Animal studies have shown that antiviral T cells are critical to immune protection against SARS‐CoV and SARS‐CoV2 infections (Zhao et al. 2010; Sun et al. 2020). In COVID19 patients, greater clonal expansion of T cells correlated with milder illness (Liao et al. 2020; Grifoni et al. 2020; Moss 2022). Similarly, immunity mediated by CD8^+^ T cells and CD4^+^ T cells against influenza virus is critical for limiting transmission and disease severity (La Gruta and Turner 2014). Thus, the antiviral function of T cells is essential for immunity against viruses, whereas dysfunction in T cells may lead to severe illness.

Despite the essential role of T cells in the immune protection against viral infection, T cells are among the immune cells most negatively impacted by aging (Zhang et al. 2021; Nikolich‐Zugich 2008; Mittelbrunn and Kroemer 2021; Lu et al. 2022). Aging is accompanied by thymic involution and a decline in the production of naïve T cells (Thapa and Farber 2019). Aging also reduces TCR repertoire and potentially generates “holes” in the defense against pathogens (Sun et al. 2022; Yager et al. 2008). In addition, the prevalence of terminally differentiated effector memory T cells and senescent T cells increases with age (Goronzy and Weyand 2017). Of note, although T cell‐mediated immune response has a high metabolic demand, aging impairs metabolic fitness of T cells (Han et al. 2023). Aging also increases the number of age‐associated T cells that express immune checkpoints including PD1 and display an exhaustion‐like phenotype (Mogilenko et al. 2021; Lee et al. 2016). Blocking PD1‐PD‐L1 interaction improves T cell surveillance of senescent cells and ameliorates aging phenotypes (Wang et al. 2022; Dahlquist et al. 2024; Majewska et al. 2024). Therefore, aging impairs T cell immunity through a variety of mechanisms. However, most studies of T cell immunosenescence focused on the baseline phenotype of bulk T cells without knowledge of antigen specificity from the aged. How aging impacts the immune response, differentiation, and molecular program of virus‐specific T cells during infection is incompletely understood.

In this study, to understand the molecular mechanism of age‐associated changes in antiviral T cell response, we used a mouse model of mouse hepatitis virus (MHV) infection. MHV belongs to betacoronavirus. Intranasal infection of mice with MHV causes SARS‐like pneumonia and lung injuries (De Albuquerque et al. 2006; Yang et al. 2014; Khanolkar et al. 2010). T cells are critical to the clearance of MHV in mice (Chua et al. 2004; Khanolkar et al. 2009). We showed a significantly higher mortality in aged mice after intranasal infection of MHV. Clonal expansion of both CD8^+^ and CD4^+^ T cells were profoundly impaired in old mice compared to young mice. Surprisingly, although aging is known to increase terminally differentiated CD8^+^ T cells at baseline, our single‐cell RNA‐seq (scRNA‐seq) analysis in aged mice after infection revealed a marked reduction in terminally differentiated effector CD8^+^ T cells and an elevated gene signature of T‐cell exhaustion. Notably, CX3CR1^+^ terminally differentiated CD8^+^ T cells, which display potent cytotoxicity in chronic viral infection and cancer (Zander et al. 2019; Hudson et al. 2019), diminished in MHV‐infected old mice. We further demonstrated that virus‐specific CD8^+^ T cells in old mice downregulated transcription factors ZEB2 and KLF2, which are critical for the development of CX3CR1^+^ terminal effector CD8^+^ T cells. In summary, our data demonstrate that aging diminishes the expansion and function of virus‐specific CD8^+^ T cells during coronavirus infection and impairs their terminal differentiation into the cytotoxic effector lineage by downregulating key transcriptional regulators.

## 2 Results

### 2.1 Aging Increases Mortality of Mice After MHV Infection

**FIGURE 1:**
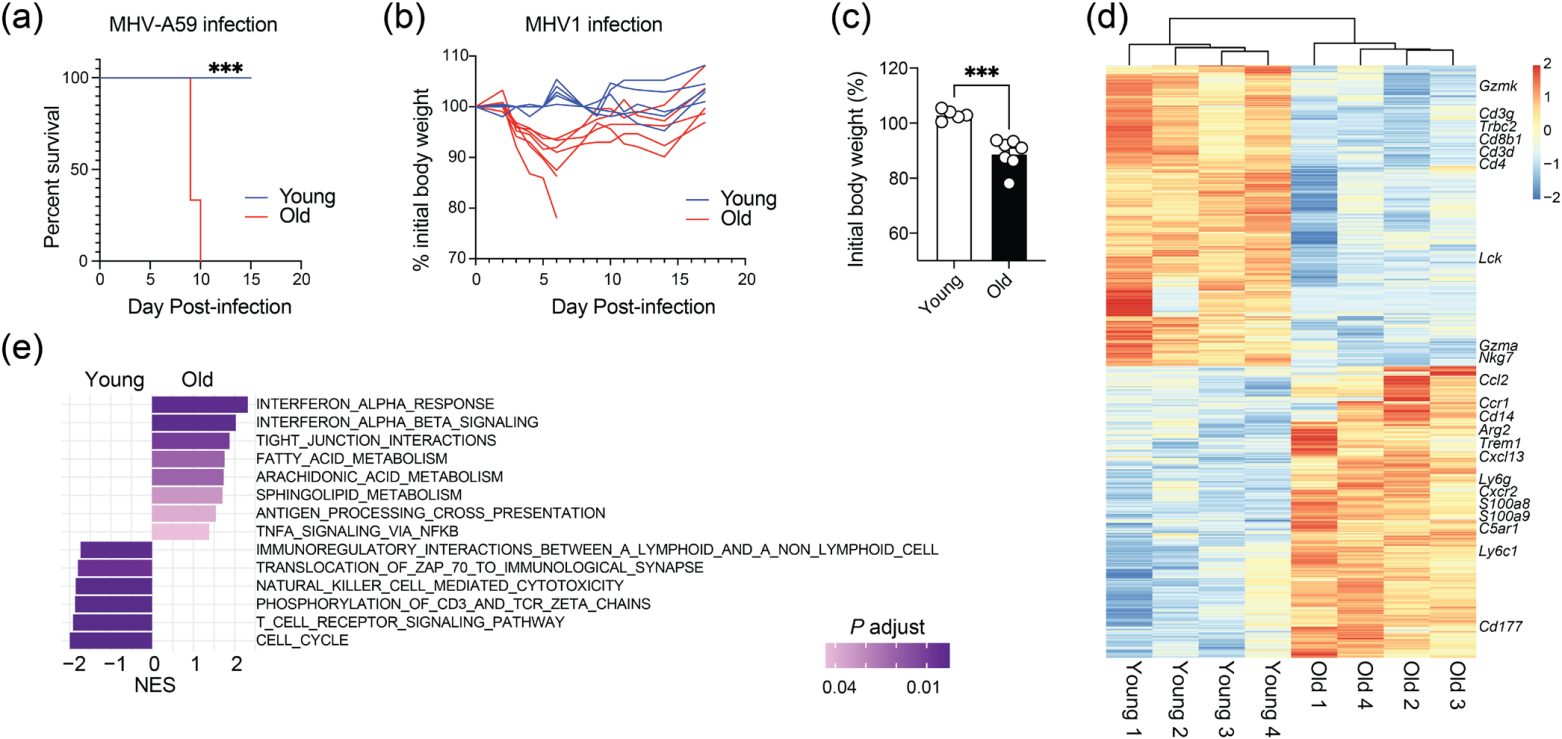
Aging promotes mortality and inflammation in the lungs after coronavirus infection. (a) Survival of young (*n* = 6 mice) and old (*n* = 6 mice) C57BL/6 mice after intranasal infection with MHV‐A59. (b, c) Body weight curve (b) and body weight on day 6 postinfection (c) of young (*n* = 5 mice) and old (*n* = 7 mice) C57BL/6 mice after intranasal infection of MHV‐1. (d, e) RNA‐seq was performed with lung homogenate from young (*n* = 4) and old (*n* = 4) C57BL/6 mice on day 7 after intranasal infection of MHV‐A59. A Heatmap of differentially expressed genes (d) and GSEA of differentially enriched pathways (e) are shown. NES, normalized enrichment score; *p*.adjust, adjusted *p* value. Data in (a–c) represent two independent experiments. Each data point represents one mouse. A log‐rank Mantel‐Cox test was used in (a). An unpaired Student's *t*‐test was used in (c). *p < 0.05; **p < 0.01; ***p < 0.001.

To evaluate how aging affects the susceptibility of mice to coronavirus infection in the respiratory tract, we intranasally infected young (2 months) and old (20 months) C57BL/6 (B6) mice with MHV‐A59. All old mice intranasally infected by MHV‐A59 died by day 10 postinfection (p.i.), whereas all young mice survived (Figure 1a). Age‐associated increase in disease severity was also observed in mice infected with another MHV strain, MHV‐1. Whereas MHV‐1 infection minimally affected the weight of young mice, the weight loss in old mice after infection reached a maximum on day 6 p.i. at around 10% of their original body weight (Figure 1b,c). The majority of MHV‐1‐infected old mice regained their body weight after the first week, whereas two old mice died in the first week after infection (Figure 1b and Figure S1a). These results suggest that intranasal MHV infection in aged mice resembles the increased disease severity seen in older adults with SARS‐CoV or SARS‐CoV‐2 infection.

### 2.2 Aging Increases Expression of Pro‐Inflammatory Genes and Pathways in the Lung After MHV Infection

To investigate the effect of aging on the antiviral response in the lung, we performed a bulk RNA sequencing (RNA‐seq) analysis with lung homogenates from young or old mice on day 7 after intranasal infection of MHV‐A59 (Figure S1b). The lung of infected old mice significantly (false discovery rate [FDR] < 0.05, fold‐change > 1.5) upregulated 634 genes and downregulated 717 genes compared with young mice. Notably, aging upregulated genes associated with tissue inflammation after infection (Figure 1d). For example, C5a complement receptor (*C5ar1*), arginase type II (*Arg2*), and TREM1 are associated with lung inflammation and damage in patients with COVID‐19 (Carvelli et al. 2020; Durante 2022; Fan et al. 2023). In addition, infected aged lung upregulated genes expressed by neutrophils, myeloid derived suppressor cells, and monocytes such as *Ly6g*, *Cd14*, *Cd177*, *S100a8*, *S100a9*, and *Ly6c1* (Figure 1d). Genes associated with chemotaxis of leukocytes such as *Cxcl13*, *Cxcr2*, *Ccr1*, and *Ccl2* were also upregulated in the lungs of infected old mice (Figure 1d). Conversely, the expression levels of genes related to T cell immune response and T cell receptor (TCR) signaling such as *Cd8b1*, *Cd4*, *Trbc2*, *Cd3g*, *Cd3d*, *Lck*, *Nkg7*, *Gzma*, and *Gzmk* were lower in infected aged lungs compared to their young counterparts (Figure 1d).

Next, we performed a gene‐set enrichment analysis (GSEA) of the bulk RNA‐seq data from infected lungs. GSEA revealed that aging increased the signature of type I interferon response, TNF signaling, antigen presentation, arachidonic acid metabolism, and sphingolipid metabolism (Figure 1e), which are involved in infection‐induced inflammation. Consistently, the levels of multiple inflammatory cytokines including IFNβ, TNFα, CCL‐2 (MCP‐1), GM‐CSF, IL‐6, IL‐12, IL‐17A, IL‐23, and IL‐27 were higher in MHV‐A59‐infected aged mice than in young mice (Figure S1c). The virus was cleared from the circulation by day 7 postinfection in all young mice and the majority of old mice (Figure S1d). In addition, pathways such as immunological synapse, TCR signaling, interaction between lymphoid and nonlymphoid cells, and natural killer (NK) cell‐mediated cytotoxicity were downregulated in the infected lung upon aging (Figure 1e). Together, transcriptomics analysis of the infected lung demonstrates an age‐associated increase in inflammation and myeloid cell recruitment and a reduction in T cell activation and T cell immune response.

### 2.3 Single‐Cell Transcriptomics Analysis of Leukocytes Infiltrating MHV‐Infected Lungs Reveals Age‐Associated Immunosuppressive Phenotype of Myeloid Cells

**FIGURE 2:**
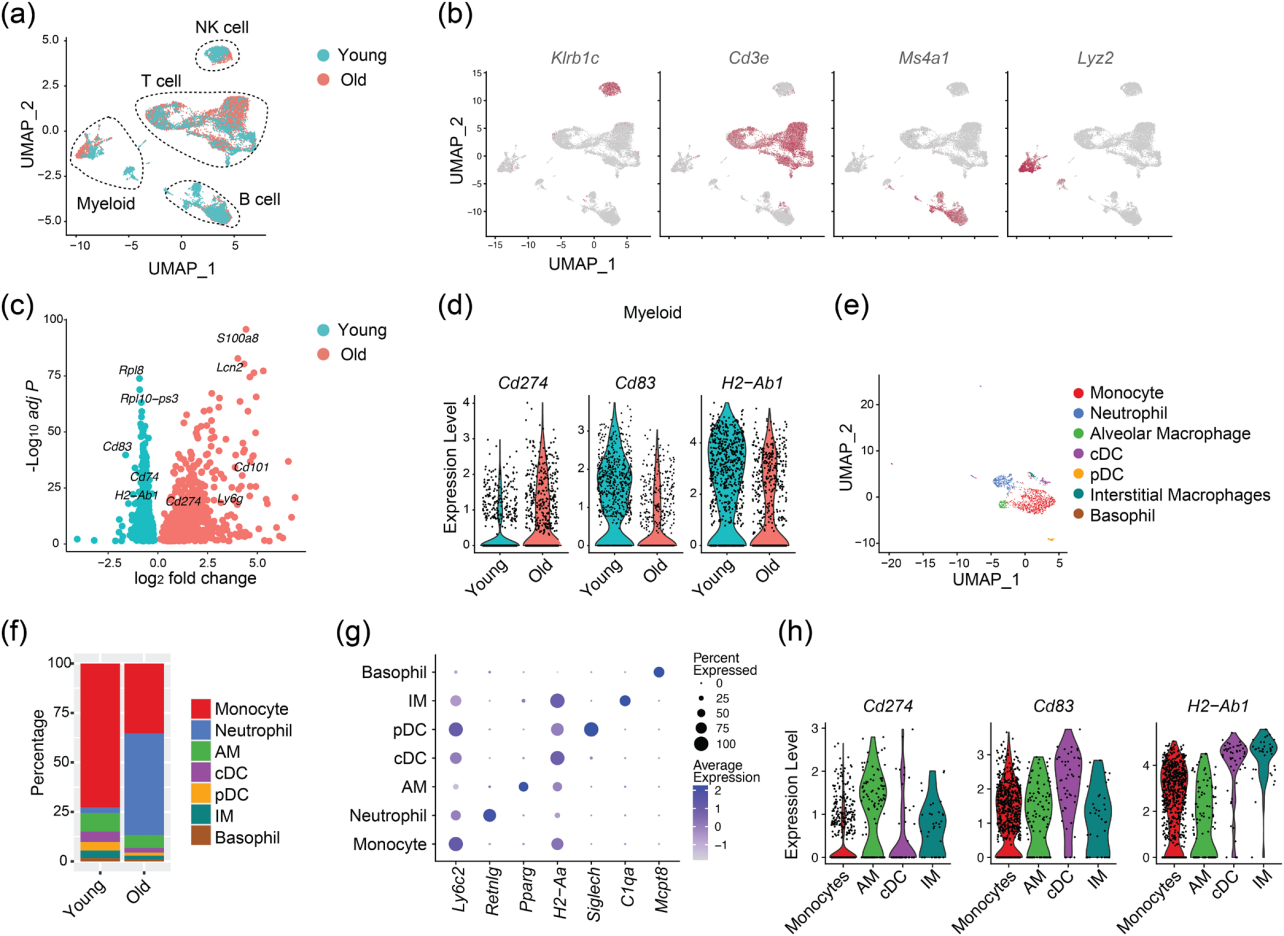
Aging dysregulates myeloid cells in the lung of MHV‐A59 infected mice. Young and old C57BL/6 mice were intranasally infected with MHV‐A59. CD45.2^+^ leukocytes were isolated and analyzed by scRNA‐seq on day 7 postinfection. (a) A UMAP plot of lung‐infiltrating leukocytes in young (teal) and old (red) mice. Major immune cell subsets are highlighted. (b) Single‐cell expression of markers associated with NK cells (*Klrb1c*), T cells (*Cd3e*), B cells (*Ms4a1*), and Myeloid cells (*Lyz2*). (c) A volcano plot shows the differentially expressed genes in lung‐infiltrating myeloid cells between young and old MHV‐infected mice. (d) Violin plots show expression of *Cd274*, *Cd83*, and *H2‐Ab1*. (e) A UMAP plot of myeloid subsets in the MHV‐infected lung including monocytes, neutrophils, alveolar macrophages (AM), conventional dendritic cells (cDC), plasmacytoid dendritic cells (pDC), interstitial macrophages (IM), and basophils. (f) The frequency of each myeloid subset defined in panel (e) in MHV‐infected young or aged lungs. (g) Dot plots of marker genes in each myeloid subset in panel (e). (h) Single‐cell expression of *Cd274*, *Cd83*, and *H2‐Ab1* in monocytes, AMs, cDCs, and IMs in the lung of MHV‐infected mice.

We showed that aging substantially alters the transcriptome of the lung after MHV infection. To gain a more granular view of how aging changes the response of different immune cell types to viral infection, we performed a scRNA‐seq with CD45^+^ leukocytes infiltrating the lungs of young and old mice on day seven after MHV‐A59 infection. A total of 7799 cells from young mice and 5318 cells from old mice were detected and projected to a Uniform Manifold Approximation and Projection (UMAP) plot (Figure 2a). Using established gene signatures of major immune cell types, we identified clusters corresponding to T cells, B cells, NK cells, and myeloid cells (Figure 2a,b). Of note, aging led to an evident shift in the UMAP space occupied by T cells and myeloid cells (Figure 2a), suggesting that aging has a greater impact on the transcriptome of these two cell types. Next, we compared the transcriptome of myeloid cells in the lung between young and aged MHV‐infected mice (Figure 2c). Notably, aged myeloid cells in the infected lung showed reduced expression of *Cd83* and *H2‐Ab1* (MHCII) (Figure 2c,d), which are markers of activated antigen presenting cells (Tze et al. 2011). In addition, the expression of *Cd274* (PD‐L1), a ligand of immune checkpoint PD1, is upregulated in aged myeloid cells (Figure 2c,d). A closer examination of lung‐infiltrating myeloid cells revealed seven subsets including alveolar macrophages, interstitial macrophages, conventional dendritic cells (cDCs), plasmacytoid dendritic cells (pDCs), monocytes, neutrophils, and basophils (Figure 2e–g). Of note, aging increased the infiltration of neutrophils to MHV‐infected lungs (Figure 2f). Next, we determined the cell types that expressed *Cd274*, *Cd83*, and *H2‐Ab1* and found that the level of *Cd274* was highest in lung macrophages whereas *Cd83* and *H2‐Ab1* were highly expressed by cDCs (Figure 2h). Therefore, scRNA‐seq of lung‐infiltrating leukocytes demonstrates a potential age‐associated shift of myeloid cells from immunostimulatory to immunosuppressive.

### 2.4 Impaired Antiviral CD8 ^+^ and CD4 ^+^ T Cell Responses in Aged Mice After MHV Infection

**FIGURE 3:**
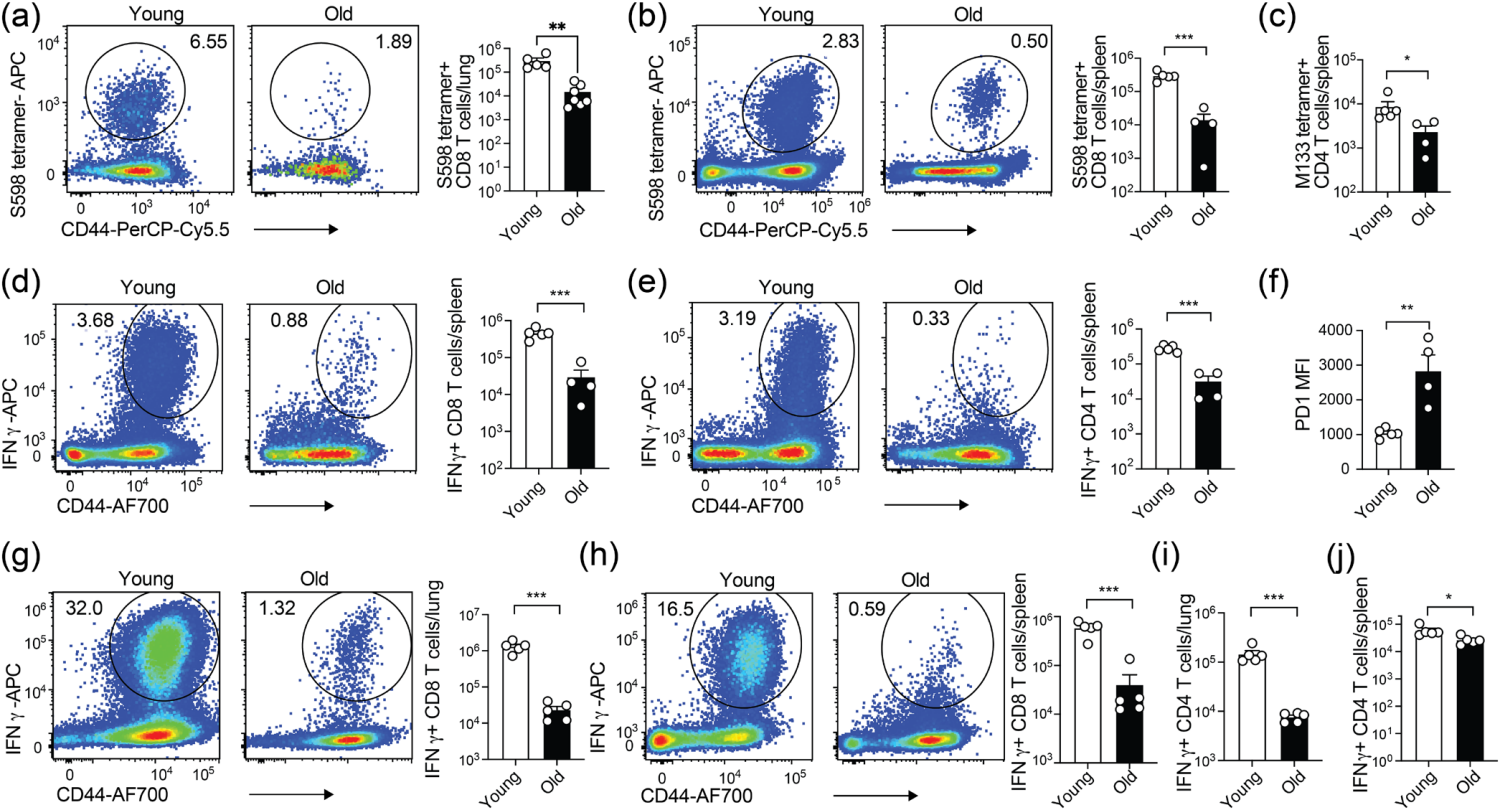
Aging impairs antiviral responses by CD8^+^ and CD4^+^ T cells against MHV. (a) Representative flow cytometry plots (left) and numbers (right) of S598 tetramer^+^ CD8^+^ T cells in the lungs of young (*n* = 5) and old (*n* = 7) C57BL/6 mice intranasally infected with MHV‐A59 on day 7 postinfection. (b) Representative flow plots (left) and numbers (right) of S598 tetramer^+^ CD8^+^ T cells in the spleens of young (*n* = 5) and old (*n* = 4) C57BL/6 mice on day 7 after intranasal infection of MHV‐A59. (c) Numbers of M133 tetramer^+^ CD4^+^ T cells in the spleen of MHV‐A59 infected young (*n* = 5) and old (*n* = 4) mice. (d, e) Representative flow cytometry plots (left) and numbers (right) of IFNγ^+^ CD8^+^ (d) and CD4^+^ (e) T cells in the spleen of MHV‐A59 infected young (*n* = 5) and old (*n* = 4) mice. (f) PD1 expression of S598 tetramer^+^ CD8^+^ T cells in the spleen of MHV‐A59 infected young (*n* = 5) and old (*n* = 4) mice. (g, h) Representative flow cytometry plots (left) and numbers (right) of IFNγ^+^ CD8^+^ T cells in the lungs (g) and spleens (h) of young and old C57BL/6 (*n* = 5 per group) on day 7 after MHV‐1 infection. (i, j) Numbers of IFNγ^+^ CD4^+^ T cells in the lungs (i) and spleens (j) of young and old MHV‐1 infected C57BL/6 (*n* = 5 per group). Data is representative of two independent experiments. Each data point represents one mouse. An unpaired Student's *t*‐test was used in (a, b, d–i). A Mann Whitney test was used in (c). *p < 0.05; **p < 0.01; ***p < 0.001; ****p < 0.0001.

T cells are essential for the control of MHV in mice (Khanolkar et al. 2009; Moss 2022). Thus, we sought to determine the impact of aging on the immune response by antiviral CD8^+^ T cells and CD4^+^ T cells in MHV‐infected mice. Young and old C57BL/6 mice were intranasally infected with MHV‐A59. On day 7 p.i., we quantified virus‐specific CD8^+^ T cells in the lung and spleen using an H2‐Kb S598‐605 tetramer corresponding to the dominant MHCI‐restricted epitope in the spike protein of MHV‐A59. There was a ~10‐fold reduction in the number of S598‐tetramer^+^ CD8^+^ T cells in both the lung and spleen of old mice compared to young mice (Figure 3a,b). To evaluate the virus‐specific CD4^+^ T cell response, we used an H2‐IAb M133‐147 tetramer corresponding to an MHCII‐restricted epitope in the membrane protein of MHV‐A59. Old mice had a much lower number of M133‐specific CD4^+^ T cells than young mice (Figure 3c). In addition, the numbers of CD8^+^ T cells and CD4^+^ T cells that produced IFNγ, an antiviral cytokine, were reduced by ~10‐fold in old mice compared with young mice (Figure 3d,e). Notably, S598‐specific CD8^+^ T cells from aged mice upregulated immune checkpoint PD1 compared to their counterparts in young mice (Figure 3f). We next sought to confirm that the age‐dependent decline in T‐cell immunity was not unique to one MHV strain. Young and old mice were intranasally infected with MHV‐1. On day 7 p.i., the number of IFNγ^+^ CD8^+^ T cells after ex vivo restimulation with S587‐594 peptide, a dominant epitope in the spike protein of MHV‐1, was ~10‐fold lower in the lung and spleen of infected old mice than those of young mice (Figure 3g,h). Similarly, the number of CD4^+^ T cells responding to restimulation by the M133 peptide was also diminished in both lung and spleen (Figure 3i,j). These results suggest that antiviral CD8^+^ and CD4^+^ T cells in aged mice were diminished in number during MHV infection.

### 2.5 Aging Induces an Exhaustion‐Like Transcriptional Signature in Virus‐Specific CD8 ^+^ T Cells Responding to MHV Infection

**FIGURE 4:**
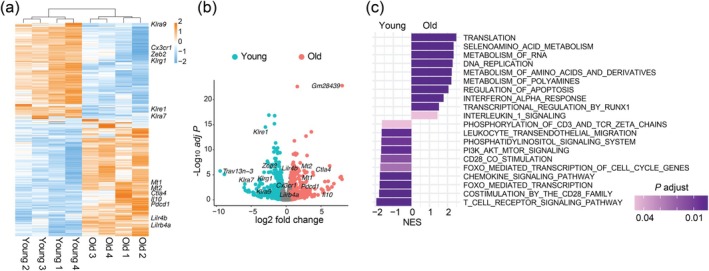
Aging induces an exhaustion‐like program in MHV‐specific CD8^+^ T cells. Bulk RNA‐seq was performed with S598 tetramer^+^ CD8^+^ T cells in young and old C57BL/6 mice (*n* = 4 mice per group) on day 7 after intranasal infection with MHV‐A59. (a, b) A heatmap (a) and a volcano plot (b) of differentially expressed genes in S598 tetramer^+^ CD8^+^ T cells between young and old MHV‐infected mice. (c) GSEA reveals differentially enriched pathways between young and old MHV‐specific CD8^+^ T cells. NES, normalized enrichment score; *p*.adjust, adjusted *p* value.

To understand the effect of aging on the transcriptional program of MHV‐specific CD8^+^ T cells, we performed a bulk RNA‐seq with S598‐tetramer^+^ CD8^+^ T cells of young and old mice on day 7 after MHV‐A59 infection (Figure S2a). MHV‐specific CD8^+^ T cells from infected old mice significantly (false discovery rate [FDR] < 0.05, fold‐change > 1.5) upregulated 326 genes and downregulated 271 genes compared to those from infected young mice. Among genes upregulated with age in MHV‐specific CD8^+^ T cells were those encoding immune checkpoints such as *Ctla4*, *Pdcd1* (encoding PD1), *Lilr4b*, and *Lilrb4a* (Figure 4a,b), which are expressed by exhausted T cells. Aging also increased the expression of *Il10*, a cytokine that suppresses antiviral immunity of CD8^+^ T cells (Brooks et al. 2006), as well as *Mt1* and *Mt2*, encoding metallothionein that promotes T cell exhaustion (Singer et al. 2017; Figure 4a,b). In addition, virus‐specific CD8^+^ T cells from old mice showed reduced expression of genes encoding killer cell lectin‐like receptors (*Klra7*, *Klra9*, *Klre1*, and *Klrg1*) as well as *Cx3cr1* and *Zeb2*.

To determine the pathways in virus‐specific CD8^+^ T cells perturbed by aging, we performed GSEA of S598‐tetramer^+^ CD8^+^ T cells from MHV‐infected young and old mice. Notably, virus‐specific CD8^+^ T cells from infected old mice showed enrichment of gene signatures of type I interferon response and apoptosis compared to those from young mice. In addition, aging downregulated gene signatures of TCR signaling, co‐stimulation, FOXO‐mediated transcription, and PI3K/AKT/mTOR signaling (Figure 4c and Figure S2b). Therefore, aging induces an exhaustion‐like transcriptional program in antiviral CD8^+^ T cells and downregulates pathways associated with T cell activation.

### 2.6 Aging Impairs the Differentiation of Antiviral CD8 ^+^ T Cells to CX3CR1 ^+^ Terminally Differentiated Effector Cells

**FIGURE 5:**
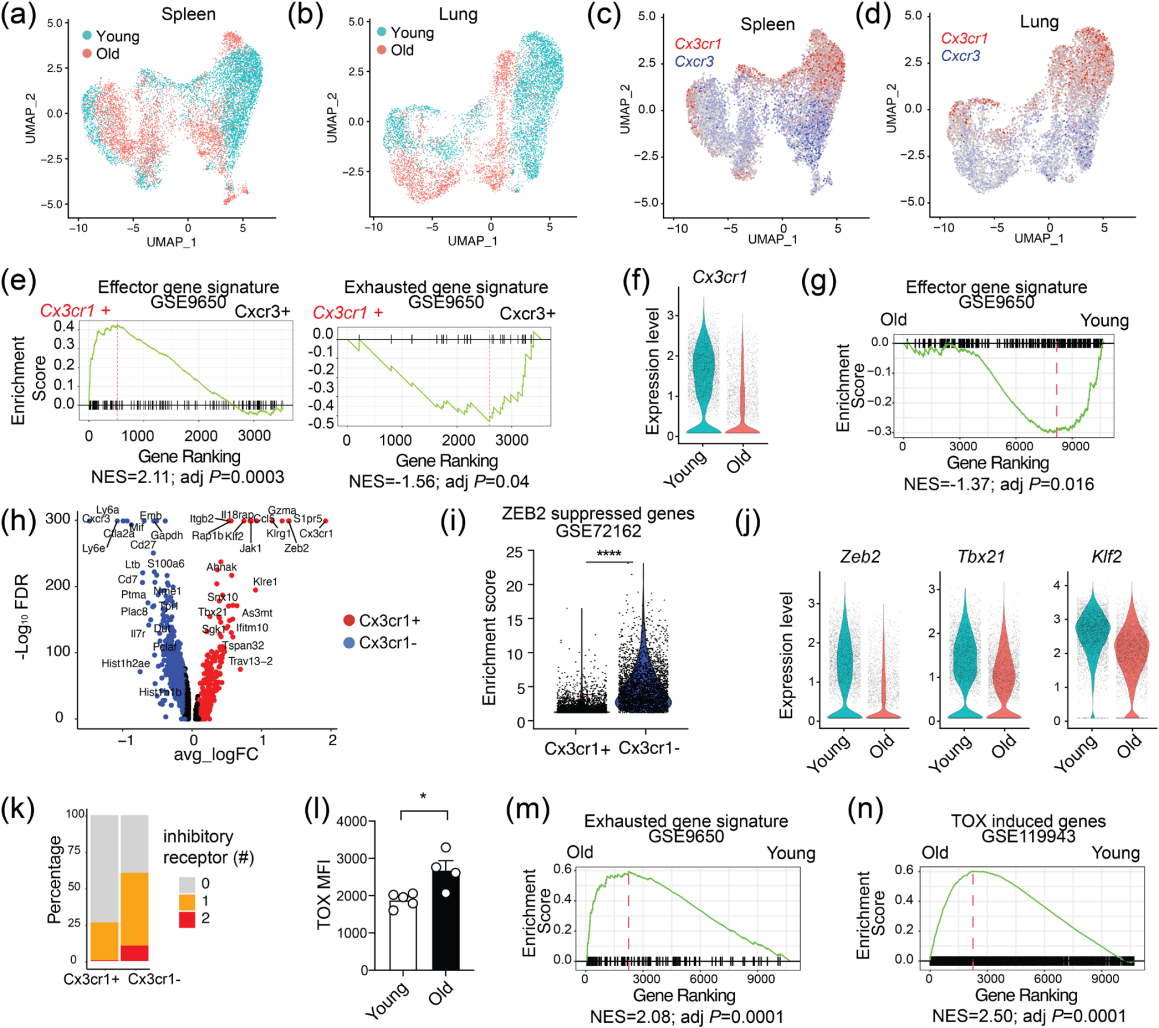
Aging impairs differentiation of effector CD8^+^ T cells in MHV infection. S598 tetramer^+^ CD8^+^ T cells collected from young and old C57BL/6 mice on day 7 after intranasal infection of MHV‐A59 were analyzed by scRNA‐seq. (a, b) UMAP plots show S598 tetramer^+^ CD8^+^ T cells in the spleens (a) and lungs (b) of young (teal) and old (red) MHV‐infected mice. (c, d) UMAP plots highlight *Cx3cr1* ^+^ *Cxcr3* ^−^ cells (red) and *Cx3cr1* ^−^ *Cxcr3* ^+^ cells (blue) among S598 tetramer^+^ CD8^+^ T cells in the spleens (a) and lungs (b) of MHV‐infected mice. (e) Enrichment of gene signatures of effector (left) or exhausted (right) CD8^+^ T cells between *Cx3cr1* ^+^ *Cxcr3* ^−^ and *Cx3cr1* ^−^ *Cxcr3* ^+^ S598 tetramer^+^ CD8^+^ T cells determined by GSEA. NES, normalized enrichment score; *p*.adjust, adjusted *p* value. (f) Single‐cell expression of *Cx3cr1* in S598 tetramer^+^ CD8^+^ T cells from MHV‐infected mice. (g) Enrichment of effector gene signature in old versus young S598 tetramer^+^ CD8^+^ T cells from MHV‐infected mice determined by GSEA. (h) A volcano plot shows the differentially expressed genes between *Cx3cr1* ^+^ *Cxcr3* ^−^ (red) and *Cx3cr1* ^−^ *Cxcr3* ^+^ (blue) S598 tetramer^+^ CD8^+^ T cells. (i) Single‐cell enrichment of ZEB2 suppressed genes in *Cx3cr1* ^+^ *Cxcr3* ^−^ (red) and *Cx3cr1* ^−^ *Cxcr3* ^+^ (blue) S598 tetramer^+^ CD8^+^ T cells. (j) Single‐cell expression of *Zeb2*, *Tbx21*, and *Klf2* in S598 tetramer^+^ CD8^+^ T cells from young (teal) and old (red) MHV‐infected mice. (k) The number of immune checkpoints expressed by *Cx3cr1* ^+^ *Cxcr3* ^−^ and *Cx3cr1* ^−^ *Cxcr3* ^+^ S598 tetramer^+^ CD8^+^ T cells. (l) TOX protein levels in S598 tetramer^+^ CD8^+^ T cells in young and old mice on day 7 after MHV‐A59 intranasal infection. (m, n) Enrichment of exhaustion gene signature (m) and TOX‐induced genes (n) in old versus young S598 tetramer^+^ CD8^+^ T cells of MHV‐infected mice determined by GSEA. An unpaired Student's *t*‐test was used in (l). *p < 0.05.

To further understand the impact of aging on the effector program of CD8^+^ T cells, we performed a scRNA‐seq analysis of S598‐specific CD8^+^ T cells in the spleen and lung of young and old mice 7 days after intranasal infection with MHV‐A59 (Figure 5a,b). In the UMAP, a large portion of aged MHV‐specific CD8^+^ T cells occupied distinct space compared to their young counterparts, suggesting that aging profoundly altered the single‐cell transcriptome of MHV‐specific CD8^+^ T cells (Figure 5a,b). In addition, scRNA‐seq data revealed that MHV‐specific CD8^+^ T cells differentiated into a *Cx3cr1* ^+^ *Cxcr3* ^−^ subset and a *Cx3cr1* ^−^ *Cxcr3* ^+^ subset after MHV infection (Figure 5c,d). GSEA revealed that the *Cx3cr1* ^+^ *Cxcr3* ^−^ subset upregulated effector T cell gene signature and downregulated exhausted T cell signature compared to the *Cx3cr1* ^−^ *Cxcr3* ^+^ subset (Figure 5e). Notably, the frequency of CX3CR1^+^CXCR3^−^ MHV‐specific CD8^+^ T cells in MHV‐infected aged mice was lower than that in young mice (Figure 5f and Figure S3a–d). Consistently, the gene signature of effector T cells was downregulated in aged MHV‐specific CD8^+^ T cells (Figure 5g). The expression level of CX3CR1 positively correlates with terminal differentiation of CD8^+^ T cells in mice and humans (Zwijnenburg et al. 2023). Therefore, aging impairs the transcriptional program of the *Cx3cr1* ^+^ terminally differentiated effector subset in CD8^+^ T cells responding to MHV infection.

Among the most upregulated genes in *Cx3cr1* ^+^ MHV‐specific CD8^+^ T cells were transcription factors *Zeb2* and *Klf2* (Figure 5h). *Zeb2* encodes a transcription repressor that promotes the expansion and terminal differentiation of effector T cells in lymphocytic choriomeningitis virus and *Listeria monocytogenes* infections (Dominguez et al. 2015; Omilusik et al. 2015). Genes repressed by ZEB2 were downregulated in *Cx3cr1* ^+^ MHV‐specific CD8^+^ T cells (Figure 5i), suggesting a role of ZEB2 in the bifurcation between the *Cx3cr1* ^+^ and *Cx3cr1* ^−^ subsets. Of note, MHV‐specific CD8^+^ T cells from infected old mice downregulated *Zeb2*, *Klf2*, and *Tbx21* compared to those from young mice (Figure 5j). *Tbx21* encodes transcription factor T‐BET that promotes effector T cell differentiation (Intlekofer et al. 2005). In addition, we and others recently showed that KLF2 promotes effector differentiation in chimeric antigen receptor (CAR) T cells and antiviral CD8^+^ T cells (Zhu et al. 2024; Fagerberg et al. 2025). Thus, this result indicates that downregulation of ZEB2, KLF2, and T‐BET in MHV‐specific CD8^+^ T cells with age may be responsible for age‐associated defects in terminal differentiation of antiviral effector T cells.

scRNA‐seq analysis revealed that cells expressing one or more immune checkpoints were enriched in the *Cx3cr1* ^−^ *Cxcr3* ^+^ subset (Figure 5k). Of note, MHV‐specific CD8^+^ T cells in infected old mice showed higher protein levels of multiple immune checkpoints such as PD1, TIM3, TIGIT, and LAG3 and a higher frequency of IFNγ^+^TNFα^−^IL2^−^ monofunctional cells compared to infected young mice (Figure S3e–g). Cell–cell communication analysis (Jin et al. 2021) between myeloid cells and MHV‐specific CD8^+^ T cells showed an age‐associated increase in ligand‐receptor interactions involving immune checkpoints, such as PGE2‐*Ptger4*, *Lgals9*‐*Havcr2* (TIM3), *Lair1*‐*Lilrb4a*, *Entpd1*(CD39)‐*Adora2a*, *Cd86/Cd80*‐*Ctla4*, and *Cd274* (PD‐L1)‐*Pdcd1* (PD1) (Figure S3h–j). We and others previously showed that transcription factor TOX established the transcriptional program of exhausted T cells (Yao et al. 2019; Alfei et al. 2019; Khan et al. 2019; Scott et al. 2019). The protein level of TOX in MHV‐specific CD8^+^ T cells was upregulated in infected aged mice compared to young mice (Figure 5l and Figure S3k,l). GSEA revealed that MHV‐specific CD8^+^ T cells in infected old mice upregulated the transcriptional program of T‐cell exhaustion (Figure 5m). In addition, aging upregulated genes activated by TOX in MHV‐specific CD8^+^ T cells (Figure 5n), suggesting an age‐associated induction of TOX‐mediated exhaustion program in MHV‐specific CD8^+^ T cells.

### 2.7 ZEB2 and KLF2 Promote Differentiation of MHV‐Specific CD8 ^+^ T Cells to CX3CR1 ^+^ Terminal Effectors

**FIGURE 6:**
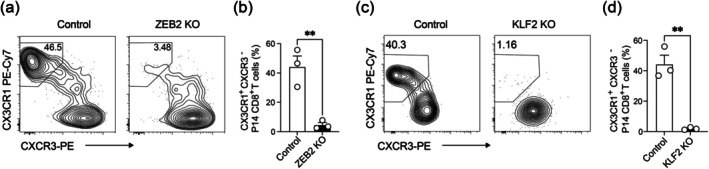
ZEB2 and KLF2 promote terminal differentiation of antiviral CD8^+^ T cells in MHV infection. (a, b) Cas9; P14 CD8^+^ T cells transduced with control or *Zeb2*‐targeting gRNA construct were transferred to C57BL/6 mice that were intranasally infected with MHV‐A59‐GFP/GP33 and analyzed on day 7 postinfection. Representative flow cytometry plots (a) and percentages (b) of CX3CR1^+^CXCR3^−^ cells in control and ZEB2 KO (*n* = 3 recipient mice per group) P14 CD8^+^ T cells are shown. (c, d) Control or *Klf2*‐targeting gRNA transduced Cas9; P14 CD8^+^ T cells were analyzed on day 7 after intranasal MHV‐A59‐GFP/GP33 infection of C57BL/6 recipients. Representative flow cytometry plots (c) and percentages (d) of CX3CR1^+^CXCR3^−^ control and KLF2 KO (*n* = 3 recipient mice per group) P14 CD8^+^ T cells are shown. Data represent two independent experiments. Each data point is from one mouse. An unpaired Student's *t*‐test was used in (b, d). *p < 0.05; **p < 0.01.

Our results above show that aging downregulates ZEB2 and KLF2 in CD8^+^ T cells responding to MHV infection. We next sought to understand the function of these two transcription factors in MHV‐specific CD8^+^ T cells. P14; Cas9 CD8^+^ T cells that express CAS9 and a transgenic TCR recognizing the GP33‐41 epitope were retrovirally transduced with a gRNA targeting *Zeb2* and transferred to C57BL/6 mice. Mice were then intranasally infected with MHV‐A59‐GFP/GP33, a recombinant MHV‐A59 that expresses GFP and the GP33‐41 epitope (Chua et al. 2004). On day 7 p.i., ZEB2 deficiency markedly reduced the frequency of CX3CR1^+^CXCR3^−^ P14 CD8^+^ T cells (Figure 6a,b). Next, we deleted KLF2 in P14; Cas9 CD8^+^ T cells by retroviral transduction with a *Klf2* targeting gRNA. Seven days after infecting recipients of P14 cells with MHV‐A59‐GFP/GP33, we observed a near complete loss of CX3CR1^+^CXCR3^−^ P14 CD8^+^ T cells caused by KLF2 deficiency (Figure 6c,d). Therefore, ZEB2 and KLF2 are essential transcriptional regulators that drive the differentiation of CX3CR1^+^ terminal effector CD8^+^ T cells during MHV infection.

## 3 Discussion

The existing paradigm that aging drives terminal differentiation of T cells is supported by studies that largely focused on the baseline phenotypic changes in T cells caused by aging. By comprehensively analyzing the differentiation and transcriptional program of virus‐specific CD8^+^ T cells responding to ongoing coronavirus infection, we show that aging suppresses terminal differentiation to CX3CR1^+^ cytotoxic effector cells and promotes exhaustion in virus‐specific CD8^+^ T cells. The age‐associated shift in the differentiation program of antiviral CD8^+^ T cells is accompanied by the downregulation of key transcription factors, ZEB2 and KLF2, that were required for the differentiation of CX3CR1^+^ cytotoxic terminal effector cells. The pro‐exhaustion transcription factor TOX and its downstream transcriptional program were upregulated in virus‐specific CD8^+^ T cells in infected old mice. Consistent with the dysregulated transcriptional program, virus‐specific CD8^+^ T cells in infected old mice showed reduced clonal expansion and antiviral cytokine production and increased expression of immune checkpoints.

It is well documented that terminally differentiated T cells accumulate with age in humans and mice. The prevalence of terminally differentiated T_EMRA_ cells progressively increases in humans during aging (Goronzy and Weyand 2017; Lu et al. 2022). In addition, CD28^−^ T cells that accumulate in the elderly are also considered terminally differentiated (Weng et al. 2009). A progressive decrease in naïve T cell pool and an increase in terminally differentiated T cells also appear in mice during aging. The terminally differentiated T cells that accumulate with age include virtual memory T cells that display an innate‐like phenotype and respond independent of antigen stimulation (Nikolich‐Zugich 2014; Goronzy and Weyand 2017; Fagerberg et al. 2025). However, whether these age‐associated changes in T cells during hemostasis also maintain when T cells respond to viral infection is less well known. Our results in mice with MHV infection show that instead of increased terminal differentiation, virus‐specific CD8^+^ T cells in old mice responding to MHV displayed impaired terminal differentiation into the CX3CR1^+^ effector lineage. Inflammatory cytokine signaling is a key driver of the development of terminal effector CD8^+^ T cells (Joshi et al. 2007). However, given the higher expression of multiple inflammatory pathways in infected aged mice, it is unlikely that the impaired terminal differentiation of aged virus‐specific CD8^+^ T cells was caused by a lack of inflammatory cytokine signaling. Our transcriptomics analysis showed that aged virus‐specific CD8^+^ T cells downregulate ZEB2 and KLF2, two transcription factors essential for the development of the CX3CR1^+^ terminally differentiated effector lineage in MHV infection. Future studies are warranted to further investigate how aging alters environmental cues and cell intrinsic pathways that govern the expression of ZEB2 and KLF2 in virus‐specific CD8^+^ T cells. The paradoxical effect of aging on T cell terminal differentiation at homeostasis and after infection is reminiscent of inflammaging and immunosenescence. One explanation is that the chronic stimulation during aging ultimately compromises the ability of T cells to adapt to acute stimuli. It is also possible that while being more prevalent in aged individuals before infection, the terminally differentiated virus‐specific T cells are outcompeted by the remaining naïve virus‐specific T cells after infection. Aging drives mitochondrial dysfunction especially in terminally differentiated T cells (Han et al. 2023), which may render them less proliferative in response to viral antigen stimulation than naïve T cells that are more frequent in young individuals. A study shows that cytotoxic effector‐like virus‐specific T cells are associated with lower TCR signaling avidity (Daniel et al. 2022). In addition, aging leads to the accumulation of terminally differentiated T cells with lower levels of costimulatory receptors and higher levels of immune checkpoints (Han et al. 2023). Therefore, despite their accumulation with age, terminally differentiated virus‐specific T cells may be outcompeted by naïve virus‐specific T cells after infection because of metabolic dysregulation, lower TCR signaling avidity, and reduced co‐stimulation. Further investigation on how the differentiation state of T cells at homeostasis impacts the immune response and differentiation of T cells after viral infection may reveal further insight into this paradox. A previous study shows that aged T cells have a different response kinetics and require stronger stimulation to be activated compared to young T cells (Pieren et al. 2019). Thus, the age‐associated alteration in effector differentiation described in our study may at least partially be due to the different response kinetics of MHV‐specific CD8^+^ T cells. Of note, aging elevated the levels of inflammatory cytokines and resulted in mortality by day 10 after MHV‐A59 infection. Given that the virus was largely cleared from the circulation even in aged mice, it is possible that unrestrained inflammation or cytokine storm may contribute to the age‐associated mortality in MHV‐infected old mice. Future investigation with different infectious doses of MHV is warranted to understand how aging impacts the kinetics of the CD8^+^ T cell‐mediated immune response.

Exhaustion is a dysfunctional state of T cells that often occurs in chronic infection and cancer and is associated with upregulation of immune checkpoints including PD1. Interestingly, T cells that show an exhaustion‐like phenotype and upregulate PD1 accumulate with age (Mogilenko et al. 2021; Lee et al. 2016). Senescent cells evade immunosurveillance via the PD1‐PD‐L1 axis and drive the aging phenotype (Wang et al. 2022; Dahlquist et al. 2024; Majewska et al. 2024). Our study shows that aging increased expression of the immune checkpoints such as PD1 and induced an exhaustion‐like phenotype in virus‐specific CD8^+^ T cells responding to MHV infection. The regulon of the transcription factor TOX, which establishes the transcriptional program of exhausted T cells (Yao et al. 2019; Alfei et al. 2019; Khan et al. 2019; Scott et al. 2019) was upregulated in virus‐specific CD8^+^ T cells in MHV‐infected old mice. Of note, myeloid cells in infected old mice expressed a higher level of *Cd274* encoding PD‐L1, suggesting that aging may suppress the antiviral immune response of CD8^+^ T cells through the PD1‐PD‐L1 axis. Although aging increases the prevalence of TOX^+^PD1^+^ CD8^+^ T cells before infection, the antigen specificity of these exhausted‐like T cells is unknown. It is also unclear whether virus‐specific CD8^+^ T cells express PD1 and TOX before encountering their cognate antigens. It is possible that aging predisposes an exhaustion‐prone state in virus‐specific CD8^+^ T cells before infection through transcriptional and/or epigenetic mechanisms. Although previous studies suggest that TOX is dispensable for CD8^+^ T cells responding to acute viral infection (Yao et al. 2019; Alfei et al. 2019; Khan et al. 2019; Scott et al. 2019), these studies were performed in young mice. The role of TOX in T cell aging may warrant further investigation. Although PD1 is expressed by exhausted T cells, the expression of PD1 in MHV‐specific CD8^+^ T cells in infected old mice does not necessarily indicate functional exhaustion. Live viruses were largely cleared from the circulation of both young and old mice on day 7 post‐infection. Nonetheless, MHV‐specific T cells in old mice could be exposed to a longer duration of antigen stimulation than those in young mice, which may lead to the upregulation of TOX and induction of an exhaustion‐like phenotype. In addition, other T cell lineages, such as tissue‐resident memory T cells and age‐associated T cells, can also express PD1 (Mogilenko et al. 2021; Yenyuwadee et al. 2022). Future investigation of the proliferative capacity and antiviral function of MHV‐specific CD8^+^ T cells in old mice is necessary to determine whether aging indeed drives functional exhaustion of antiviral CD8^+^ T cells. We demonstrated that aging increased the levels of multiple inflammatory cytokines including type I interferon and TNFα and enhanced recruitment of neutrophils in infected old mice. Previous studies have shown that excessive type I interferon and TNFα suppress antiviral T cell immunity (Suresh et al. 2005; Snell et al. 2017; Wu et al. 2016). In addition, neutrophils can also suppress T cell immunity (Bert et al. 2023). Thus, in addition to T cell exhaustion, other mechanisms such as elevated inflammation and enhanced neutrophil recruitment may contribute to the impaired antiviral T cell immune response in MHV‐infected old mice.

Together, our study elucidates the effect of aging on the differentiation program of virus‐specific CD8^+^ T cells responding to coronavirus infection and sheds light on the potential mechanism of age‐associated dysregulation of antiviral T cell immunity.

## 4 Experimental Procedures

### 4.1 Mice

Young (2 months) and old (18–22 months) female C57BL/6 mice were acquired from National Institute of Aging aged rodent colonies. All animal experiments were conducted in accordance with protocols approved by the Institutional Animal Care and Use Committee at the University of Texas Southwestern Medical Center and University of Colorado School of Medicine.

### 4.2 Viruses and Infections

The original MHV‐A59, MHV‐A59‐GFP/GP33, and MHV‐1 stock were provided by Dr. Julian L Leibowitz (Texas A&M University), Dr. Susan Weiss (University of Pennsylvania), and Dr. Tanya Miura (University of Idaho), respectively. Viruses were propagated in 17Cl‐1 cells, which were provided by Dr. Tanya Miura (University of Idaho). Viruses were stored at −80°C. 17Cl‐1 cells were cultured in DMEM medium with 10% FBS, 2 mM glutagro, and Penicillin–Streptomycin. Virus titer was determined by plaque assay using L2 cells as previously described (Leibowitz et al. 2011). For infections, mice were anesthetized and intranasally infected with 5 × 10^6^ plaque‐forming unit (PFU) MHV‐A59 or 10^5^ PFU MHV‐1.

### 4.3 Tissue and Cell Isolation

Spleens were minced to a single‐cell suspension. Red blood cells were removed by incubating cells with ACK Lysing Buffer at room temperature for 2 min. After adding additional RPMI medium with 2% FBS, leukocytes were collected by centrifugation and filtered with 70uM cell strainers. Lungs were cut into fine pieces and digested with Liberase TL (Sigma, # 05401020001) at 37°C for 40 min within a shaking Incubator. Digested cells and fragments were forced through a cell strainer in RPMI medium with 2 mM EDTA to generate a single‐cell suspension. After centrifugation, cells were resuspended in 44% Percoll (Cytiva #17‐0891‐01) diluted with RPMI, underlaid with 67% Percoll diluted with PBS, and centrifuged at 2000RPM for 20 min at room temperature with acceleration at 9 and deceleration at 0. Interfaces containing leukocytes were collected after centrifugation.

### 4.4 Flow Cytometry and Sorting

For flow cytometry staining, cells were first blocked with PBS buffer with 2% BSA and 2 mM EDTA for 10 min at 4°C and then stained with antibodies and tetramers at 4°C for 20 min in PBS buffer with 2% calf serum. The following antibodies and dyes were used for flow cytometry and sorting: CD8a PerCP‐Cy5.5 (53–6.7), CD4 PerCP‐Cy5.5 (RM4‐5), CD44 Alexa Fluor 700 (IM7), PD1 PE‐Cy7 (RMP1‐30), IFNγ (XMG1.2), CX3CR1 PE‐Cy7 (SA011F11), CXCR3 (CXCR3‐173), LIVE/DEAD Fixable Near‐IR Dead Cell Stain Kit, and LIVE/DEAD Fixable Aqua Dead Cell Stain Kit were purchased from Thermo Fisher or Biolegend. H2‐Kb S598‐605 (RCQIFANI) tetramer and H2‐IAb M133‐147 (TVYVRPIIEDYHTLT) tetramer were acquired from the NIH Tetramer Core Facility. For measuring cytokine production, cells were incubated with the corresponding peptide, monensin (Biolegend) and brefeldin A (Biolegend) at 37°C for 5 h in a tissue culture incubator. After staining surface markers, cells were permeabilized and fixed with BD Cytofix/Cytoperm Fixation/Permeabilization Kit (#554714). Cells were then incubated with antibodies against intracellular markers in BD Perm/Wash buffer (#554723). Staining of transcription factors was performed with eBioscience Foxp3/Transcription Factor Staining Buffer Set (#00‐5523‐00).

### 4.5 Multiplex Cytokine Assay

Plasma was collected from mice on day 7 after MHV‐A59 infection. The levels of IL‐23, IL‐1α, IFNγ, TNFα, CCL2 (MCP‐1), IL‐12p70, IL‐1β, IL‐10, IL‐6, IL‐27, IL‐17A, IFN‐β, and GM‐CSF were measured with LEGENDplex Mouse Inflammation Panel (13‐plex) (Biolegend, #740446) according to the manufacturer's manual.

### 4.6 Sample Preparation for RNA Sequencing

Lung fragments from four biological replicates per group were placed in QIAzol Lysis Reagent (QIAGEN) and homogenized. Total RNA was extracted using the miRNeasy Micro Kit (QIAGEN). We FACS‐sorted 50,000 S598 tetramer^+^ CD8^+^ T cells from four biological replicates per group and stored them in QIAzol Lysis Reagent. Total RNA was then extracted using the miRNeasy Micro Kit (QIAGEN). For RNA from both lung fragments and T cells, mRNA was isolated using the Poly(A) mRNA Magnetic Isolation Module (NEB), and RNA‐seq libraries were prepared with the NEBNext Ultra II RNA Library Prep Kit for Illumina (NEB). The multiplexed libraries were sequenced on a NovaSeq 6000 using a 50‐bp paired‐read configuration, achieving a minimum of 20 million reads per sample.

### 4.7 Sample Preparation for Single‐Cell RNA Sequencing

We FACS‐sorted cells from at least three mice per group. After a PBS wash, the cells were loaded onto a chromium single cell chip G to generate gel bead‐in‐emulsions (GEMs). Barcoded DNA was extracted from the GEMs, amplified, and purified to construct gene expression (GEX) libraries according to the chromium single cell 5′ Library Construction Kit (10X) protocol. The multiplexed GEX libraries were sequenced on a NovaSeq 6000 with a read configuration of 26 Bp (Read 1) × 10 Bp (i7 Index) × 10 Bp (i5 Index) × 90 Bp (Read 2), achieving a depth of 30,000 reads per cell.

### 4.8 Data Analysis for RNA‐Seq

Sequence reads from each RNA‐seq library were aligned to the mouse genome (mm10) using STAR (v2.5.4). Transcript abundances were quantified with Kallisto (v0.44.0). Differentially expressed genes were identified using EdgeR (v4.2.2) with an FDR < 0.05 and a fold change > 1.5. Gene set enrichment analysis was conducted using clusterProfiler (v4.12.6), and heatmaps were generated with Pheatmap (v1.0.12).

### 4.9 Data Analysis for scRNA‐Seq

Sequence reads from each scRNA‐seq library were mapped to the mm10 genome using Cell Ranger (v6.0.0). The UMI vs. cell count matrices generated in this step were used for downstream analysis with Seurat (v4). Cells in the top 2% or bottom 2% for detected genes, as well as those with more than 5% of genes mapped to mitochondria, were excluded from the analysis. Cells isolated from old and young mice in the same batch were merged. Following log‐normalization, the effects of cell cycle, number of detected genes, and percentage of mitochondrial genes were regressed out. The top 2000 variable genes were used for principal component analysis (PCA). Cell clustering and UMAP embedding were performed based on the top 20 principal components. Differentially expressed genes were identified using the FindMarkers function (min.pct = 0.1, logfc.threshold = 0.1). Gene set enrichment scores were calculated using Fisher's exact test as previously described (Yao et al. 2019). The R package CellChat (version 2.1.2) was employed to infer and analyze cell–cell communication networks across various cell populations by integrating single‐cell RNA sequencing data with the pre‐existing ligand‐receptor interaction database CellChatDB (Jin et al. 2025). Briefly, the expression matrix and cell type information of B cells, myeloid cells, NK cells, and MHV‐specific T cells in the lung of young and old mice on day 7 after intranasal infection of MHV‐A59 were imported into CellChat. The overall probability of cell–cell communication among these populations was assessed using the computeCommunProb function, which calculates the likelihood of interactions based on the ligand‐receptor pairs. The rankNet function was used to identify the conserved and context‐specific signaling pathways in young and old mice.

### 4.10 Statistical Analysis

Statistical analysis was performed with Prism (GraphPad, v10.4.0) and R (v4.1.3). Differences between two experimental groups were analyzed with a two‐tailed Student's *t*‐test. *p* Value < 0.05 was considered statistically significant.

## Author Contributions

T.W. supervised the project and designed the study. Z.Z., G.L., Y.L., K.Y., J.H., J.A.D., B.M.E., C.Y., and T.W. performed the experiments and analyzed the data. T.W. and C.Y. wrote the manuscript. All authors have reviewed and approved the manuscript.

## Conflicts of Interest

The authors declare no conflicts of interest.

## Supporting information


Figure S1.

## Data Availability

Bulk RNA‐seq and scRNA‐seq data generated in this study is available at Gene Expression Omnibus (GSE284243, GSE284244).
