# Supplementary material for: Aging Compromises Terminal Differentiation Program of Cytotoxic Effector Lineage and Promotes Exhaustion in CD8 + T Cells Responding to Coronavirus Infection
Source: Aging Cell. 2025 May 21;24(8):e70109. doi: 10.1111/acel.70109 (PMC12341792; doi:10.1111/acel.70109)
Supplement: Supplementary file 1 — Figure S1. [file ACEL-24-e70109-s001.docx]

**
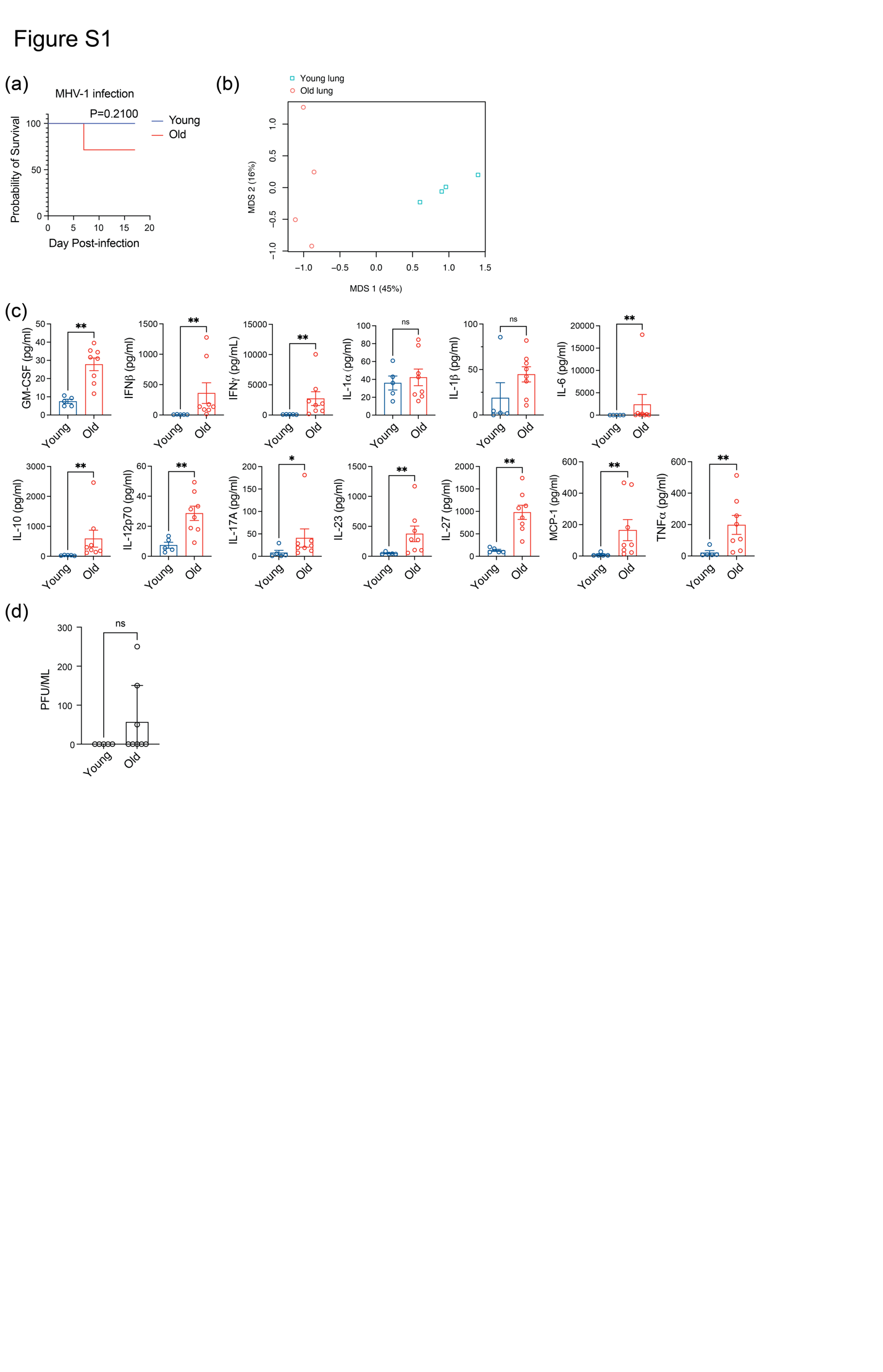
SUPPLEMENTARY FIGURE 1** (a) Survival of young (n=5) and old (n=6) C57BL/6 mice after intranasal infection with MHV-1. (b) Multidimensional scaling (MDS) analysis of RNA-seq performed with lung homogenate from young and old mice after MHV-A59 infection as in **FIGURE 1d**. (c) Multiplex measurement of inflammatory cytokines in the plasma of young (n=5) and old (n=8) C57BL/6 mice on day 7 after intranasal infection of MHV-A59. (d) Plasma viral titers in young (n=5) and old (n=8) C57BL/6 mice on day 7 after intranasal infection of MHV-A59. Data in (a, c, d) represent two independent experiments. A log-rank Mantel-Cox test was used in (a). A Mann Whitney test was used in (c, d). **P* < 0.05; ***P* < 0.01.

**
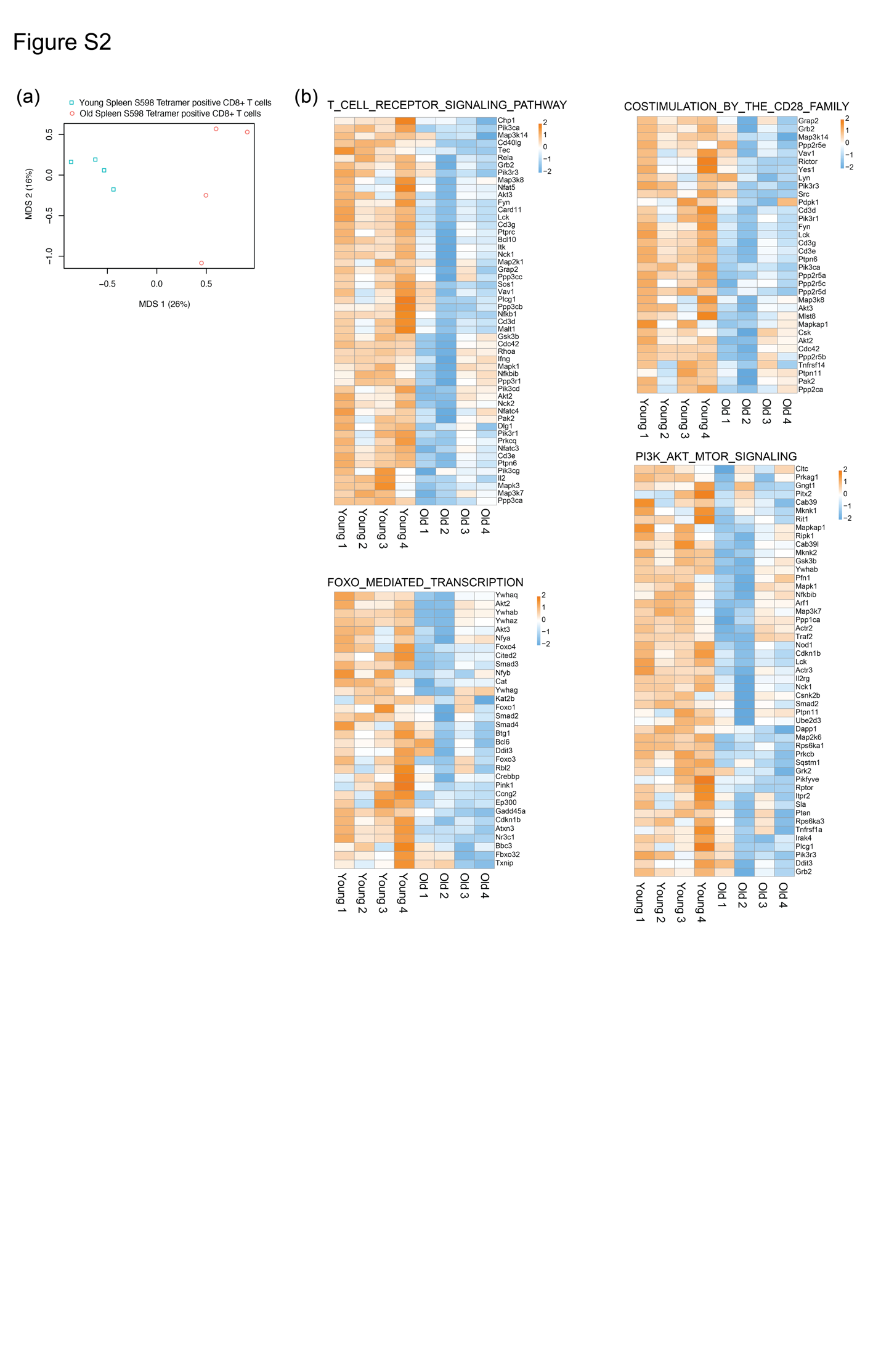
SUPPLEMENTARY FIGURE 2** (a) MDS analysis of RNA-seq data from S598 tetramer^+^ CD8^+^ T cells in MHV-infected young and old C57BL/6 mice as in **FIGURE 4**. (b) Heatmaps of top genes in each selected enriched pathway determined by the GSEA in **FIGURE 4c**.

**
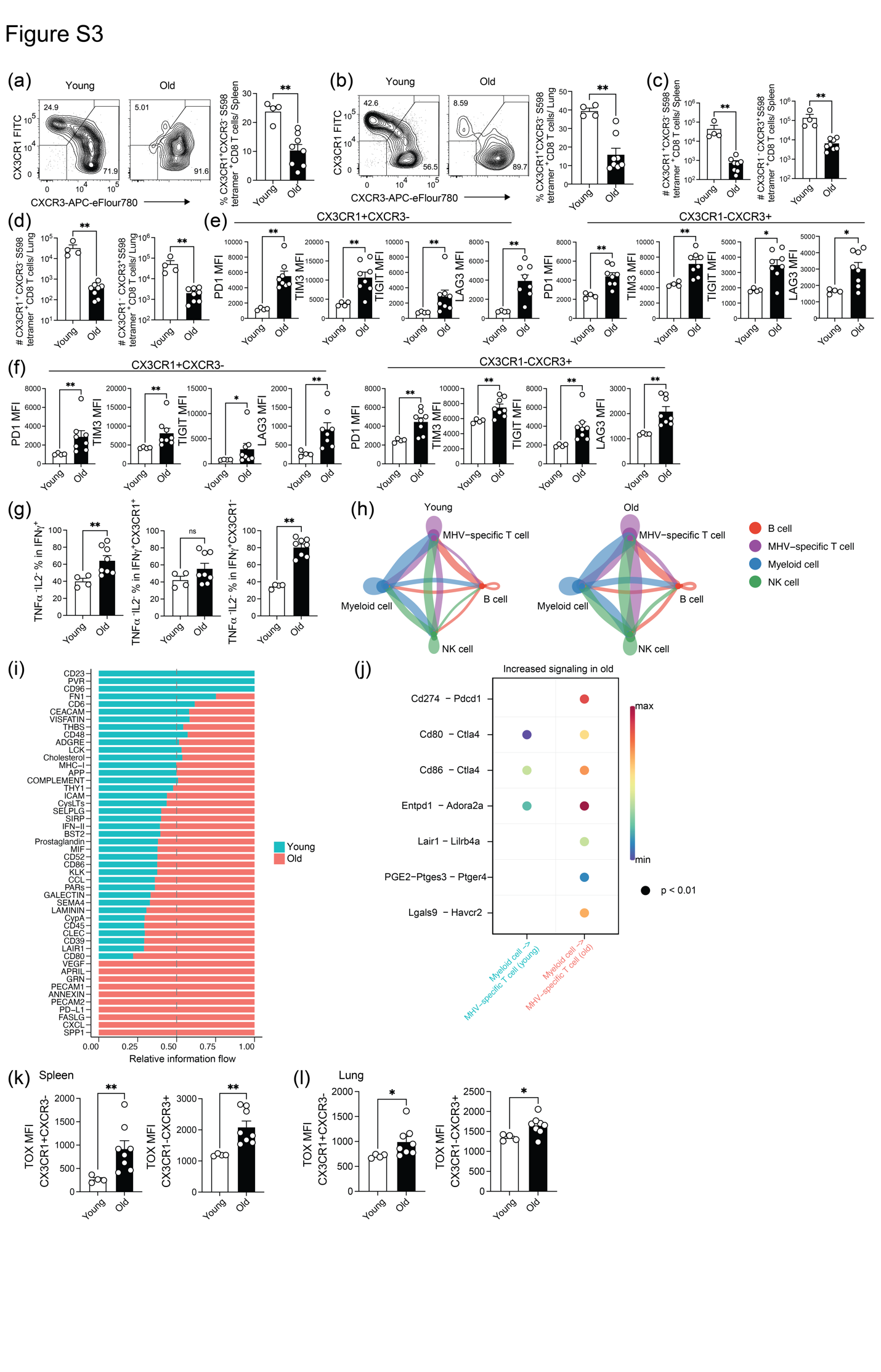
SUPPLEMENTARY FIGURE 3** (a-d) The frequencies (a, b) and numbers (c, d) of CX3CR1^+^CXCR3^-^ and CX3CR1^-^CXCR3^+^ S598 tetramer^+^ CD8^+^ T cells in the spleen (a, c) and lung (b, d) of young (n=4) and old (n=8) C57BL/6 mice on day 7 after MHV-A59 intranasal infection. (e, f) The levels of PD1, TIM3, TIGIT and LAG3 in CX3CR1^+^CXCR3^-^ and CX3CR1^-^CXCR3^+^ S598 tetramer^+^ CD8^+^ T cells in the spleen (e) and lung (f) of MHV-A59-infected young (n=4) and old (n=8) C57BL/6 mice on day 7 post-infection. (g) The frequencies of IFNγ^+^TNFα^-^IL2^-^ CD8^+^ T cells in the spleen of MHV-A59-infected young (n=4) and old (n=8) C57BL/6 mice on day 7 post-infection after 5-hour stimulation with S598 peptide. (h-j) Cell-cell communication analysis by integrating the scRNA-seq data in **FIGURE 2** and **FIGURE 5**. The number of interactions among MHV-specific CD8^+^ T cells, myeloid cells, NK cells, and B cells in MHV-A59-infected young and old mice (h), the relative information flow in young and old mice (i), and interactions between MHV-specific CD8^+^ T cells and myeloid cells that increased in old mice (j) are shown. (k, l) TOX protein levels in CX3CR1^+^CXCR3^-^ and CX3CR1^-^CXCR3^+^ S598 tetramer^+^ CD8^+^ T cells in the spleen (k) and lung (l) of MHV-A59-infected young (n=4) and old (n=8) C57BL/6 mice on day 7 post-infection. Data in (a-g, k, l) is representative of two independent experiments. Each data point represents one mouse. A Mann Whitney test was used in (a-g, k, l). **P* < 0.05; ***P* < 0.01.
